# Jury Trial of the Pauper Insane

**Published:** 1886-10

**Authors:** 


### Editorial Department.

F. E. DANIEL, M. D., EDITOR AND PUBLISHER.

COLLABORATORS:

*E. J. Doering, M. D., Chicago.*
*Geo. Cupples, M. D., San Antonio.*
*Odo Betz, M. D., Germany.*
*E. J. Beall, M. D., Fort Worth, Texas.*
*T. C. Osborn, M. D., Cleburne, Texas.*
*Wm. Penny, M. D., New York.*
*H. O. Marcy, M. D., Boston.*
*C. K. Gregg, M. D., Mexico.*
*R. M. Swearingen, M. D., Austin.*
*C. H. Wilkinson, M. D., Galveston.*
*J. W. McLaughlin, M. D., Austin.*
*R. H. L. Bibb, M. D., Mexico.*

### Editorial.

### JURY TRIALS OF THE PAUPER INSANE.

WHILE the medical profession of Texas is, and for some years has been zealously engaged in the laudable endeavor to procure the enactment of a sensible and efficient law to "regulate the practice;" or, rather, to restrict the privilege of practicing medicine to the hands of *physicians*, the fact is overlooked that there are other matters in which medical men are interested, and which, affecting the welfare of a large class of unfortunates, call for reform as loudly, and as urgently, as that of the promiscuous practice of medicine.

The statute laws of Texas, and of other States which require a jury trial of an insane pauper, as a precedent necessary to his admission into an asylum, are not only impracticable and attended with many absurdities, but, according to Dr. D. R. Wallace, State Superintendent of Asylum at Terrell, they are absolutely harmful; and, to use his own words, "can be shown to be contrary to humanity and common sense." Medical men alone can comprehend these things, and intelligent legislation can be effected only through their advice, or, at least, by their co-operation. They are not only "interested" in this matter, as just remarked, but there is a dreadful responsibility involved in their *not* procurring the passage of such laws as will mitigate the sorrows, and render less miserable the lot, of the unfortunates among us—prisoners, the blind, the deaf mutes, and the insane. The State provides bountifully for its unfortunates, yet for the want of intelligent legislation, in some instances, those for whom the asylum comforts were intended and provided, are deprived of them; or, as in the case under discussion, receive them only in such a way as to make them a curse rather than a blessing.

This state of things may fairly be attributed—as well as many other sins of omission—to the want of a State Board of Health. It does seem strange that an intelligent people will attempt to legislate on medical and sanitary matters—will endeavor to protect the public health and to provide for the unfortunate classes—without even consulting the only men or body of men who know anything whatever about the requirements of the case. But, that is a threadbare subject; and the hope is Utopian that the people of Texas will ever enjoy the benefience of intelligent sanitary laws enacted by the advise of those learned in medicine.

Dr. Wallace is a man extensively learned in psychological medicine as well as in medical jurisprudence. He has had large experience with diseases of the mind, and has closely observed insanity in all its phases. He is at present in charge of the large branch State Lunatic Asylum at Terrell, and was formerly in charge of the original and main Asylum at Austin. His long connection with the asylums, his extensive learning in that peculiar field of study, and his knowledge of the practical working of the laws concerning the insane, render him peculiarly fitted to speak of the needs of these unfortunates and the necessity of reforms.

In a paper before us, a reprint from the Transactions of the Texas State Medical Association for 1886,* (now issuing

* "Some Reflections on Medical Ethics, Medical Legislation, and Jury Trials of the Insane," by D. R. Wallace, M. D., L.L. D., Terrell, Texas, read before the Texas State Medical Association, Dallas, April, 1886.

from the office of the Secretary) Dr. Wallace draws a sad, sad picture of the working of the laws governing the admission of pauper insane into the asylums. He calls upon the medical profession, and especially upon his confreres of the State Medical Association, *to assist him* in procuring the repeal of this obnoxious law, or such modification of it as will strip it of its baleful influences on the poor unfortunates, and substitute therefore such clause as will effectually secure to them the benefits intended by the humane, but not advised framers of the law, and the generosity of the State in such provision. He calls on the State Association to "give him a lift." He intends, we infer from the paper, to make an effort at such repeal or modification at the next session of the Legislature; but, bearing in mind, as we do, the fate of other efforts at legislation on behalf of the State Association, we do not clearly *see how* the Association can do anything. If the Legislature would give us a State Board of Health, it would be in keeping with the dictates of common sense to listen to the advice of such Board; indeed, a State Board of Health should be the source from which all medical and sanitary enactments by the Legislature originate; it should be an *advisory board*. How do legislators make laws for the farming interests, or any other branch of industry, without a knowledge of the necessities of the case, derived from those who have practical experience? It is a fact that all other classes are consulted as to the needs of protection or reform, except medical men as to sanitation!

Dr. Wallace says: He has always found the Legislature of Texas ready and willing to do anything reasonable in regard to this unfortunate class, but he evidently anticipates trouble: "Judging," he says, "from what has taken place in the State of Illinois—the only other State, I believe, in which a similar provision prevails—it may not turn out to be so easy a matter," [and, he might have said, judging from what took place in Texas at the last session of the legislature—when they laughed to scorn the bill to regulate the practice, and refused to pass the one to create a State Board of Health because "Texas was not *able* (?) *to afford* the appropriation of $3,000," called for to carry out its provisions.—ED.]

"It is a curious fact," continues the author, that "there [in Illinois] superintendents of insane hospitals, boards of charity, and others, have been laboring for its repeal for years."

It must be borne in mind that the law in Texas does not apply to pay patients—only to paupers—and, therefore, all this circumlocution preliminary to placing a person in an asylum is unnecessary. It has been urged that it is necessary, in order to prevent abuse; to prevent interested persons from getting possession of the property of the accused. This does not apply to paupers, therefore Dr. Wallace does not discuss that objection; but, with reference to other features, he says:

"A much more serious objection to jury trials is found in the delay incident to such legal process. I have known the friends of an insane person to put off from day to day, and from week to week, until months had passed, before the unfortunate could be gotten to the hospital; and it must be added, this was precisely the time during which, if anything could have been done at all, it must have been attempted. In the meantime, the patient passed into chronic mania, whence he subsides rapidly into dementia. * * * A degredation from our mental and emotional eminence in creation, to a state of permanent mindlessness, in which one is dead to the love and hatred, and to the joys and pains of life—oblivious of the past and unconcerned for the future; stirred by no ambition; capable of no effort; and unmoved by any motive—a mere human hulk, like Byron's Bark—

'Thrown, when the war of winds is o'er,
A solemn wreck on fortunes shore,
'Mid sullen, calm and silent bay
Unseen, to drop by dull decay!' "

The author then draws a picture of the operation of this evil law, as follows:

"Take an imaginary case. It cannot be made as dramatic as the real ones, many of them. Remember two traits common to nearly all insane people; they do not consider themselves insane; they are incredulous and suspicious—more so, generally, toward their friends and relations than toward strangers. An insane person is induced to take a ride—is induced to prolong it to a county town. Arrived here, he is inveigled into the court house. He is at once confronted by judge, jury and lawyers. These he is accustomed to associate with trials and criminal prosecutions. He soon learns *he* is the subject of inquiry—that his conduct is being investigated; hears his name, understands witnesses giving evidence in regard to his conduct. If he comprehends the charge, that he is insane, and it is rarely he does so— he *knows* it is not so; *knows* his friends *know* it is not so; thinks it is a mere pretense; the true cause or charge is kept out of sight. He comes out of the court house amazed, dazed and alarmed. He wants to leave the country; his friends propose a trip—to some watering place, it may be—some eastern city; Europe, perhaps. He consents, and starts upon his journey; he stops at Austin or Terrell; takes a cab; is driven up to a large building. Asking what it means, is told it is a fine, first-class hotel, where they will get dinner, and, perhaps, rest over night. They enter. He (the patient), at the end of this eventful farce, finds himself locked up in a lunatic asylum. He at once wakes up to the reality—to feel he has been deceived, outraged, foully dealt with. Better, infinitely better, to have told him the truth from the first; told him he was sick, crazy—anything true—so as not to have deceived him, even if it had resulted in having to take him to the asylum by force. Never deceive a crazy man; better, far better, put him in chains. * * * * Now, ask him what he is doing there. He answers, "I don't know. They tried me, and have sent me here to prison. They say I am crazy. I know I am not, and I know they know I am not. I have done nothing to be imprisoned for.

"That he has been deceived, he knows, and he very naturally concludes that the officers of the asylum, in whose custody he is placed, have had a hand in it—are, in fact, the prime movers."

Dr. Wallace, it seems, would prefer that the commitment to the custody of the asylum should be "upon the certificate of two responsible practitioners of medicine," and says it may safely be assumed that there is no more risk to personal liberty in this way than upon the verdict of a jury. Very true, as regards paupers, but still we do not see how an insane person can be gotten into an insane asylum without deceiving him in some way.

We sincerely hope the Doctor may be able to make the Legislature see the matter in the proper light, and to so modify the law as to render it more compatible with the dictates of humanity, and with the welfare of the poor creatures for whose benefit (?) it was enacted. And we would also be glad to see the State Medical Association take some action in the matter, at least, to declare their sense of the necessity for reform, as requested by Dr. Wallace.

